# Cancer incidence, mortality, and survivorship in African women: a comparative analysis (2016–2020)

**DOI:** 10.3389/fgwh.2023.1173244

**Published:** 2024-01-11

**Authors:** Chibuikem Chrysogonus Nwagwu, Uchenna Petronilla Ogoke

**Affiliations:** Department of Mathematics and Statistics, University of Port Harcourt, Port Harcourt, Nigeria

**Keywords:** relative survival rate, mortality-to-incidence ratio, cancer survivorship, cancer prevalence, life tables

## Abstract

**Objectives:**

This research aims to provide concrete insight into cancer incidence, mortality, and survivorship dynamics among African women between 2016 and 2020.

**Methods:**

The study computes the Mortality-to-Incidence Ratio (MIR) for 53 countries in Africa with available mortality and incidence data. It uses relevant Life Tables to obtain the 5-year Relative Survival rate for women in different age cohorts based on General Survival Rate and 5-year Cancer Prevalence data from the World Health Organization (WHO). The study performs an analysis of variance tests.

**Results:**

The results of the initial data analysis show that women in the top economies in Africa have the highest cancer incidence and mortality. The study also finds that women in Northern and Southern African countries have higher relative survival rates and lower MIR than other African regions. ANOVA results confirm statistically significant differences in 5-year relative survival across the African regions. The relative survival at 5 years was an average of 45% across all age groups within the continent although relative survival is highest among females aged 5–19 and 80–84. The lowest relative survival rates are seen for infants (0–4), adolescents and young adults (25–29), and the very elderly (85+).

**Conclusion:**

The study concludes that while cancer incidence in Africa is linked to affluence, survival is very challenging, especially for the least developed economies in Western, Eastern, and Central Africa. The results indicate the need for crucial intervention in the continent concerning awareness, research, and data collection methodology.

## 1 Introduction

Nearly 10 million people died from cancer in 2020, making cancer the primary cause of death globally (1). Within two decades, the share of deaths from non-communicable diseases (NCDs) like cancer in Africa has risen from 24% to about 37% (2) with several notable ones affecting women, including breast cancer, colorectal cancer, endometrial cancer, lung cancer, cervical cancer, skin cancer, and ovarian cancer (3). By the next decade, Africa will dominate globally as NCDs will become the leading cause of death over communicable diseases by 2030 (4).

Carcinogens are substances that have the potential to cause cancer. They can be found in various forms, such as in the air, certain products, or chemicals present in food and beverages (5). Studies have identified several substances, including radon, tobacco, asbestos, crispy or brown foods, formaldehyde, ultraviolet rays, alcohol, processed meat, motor exhaust, and pollution, that can contribute to cancer development if consumed excessively (6). However, it is essential to understand that not everyone exposed to carcinogens will necessarily develop cancer. Factors like individual susceptibility, which can be influenced by genetics and other personal circumstances, play a role in determining the likelihood of developing cancer (5, 6). Smoking, alcohol consumption, unhealthy diet, and physical inactivity leading to obesity, breastfeeding, and air pollution are some risk factors for cancer and other non-communicable diseases considered in this work (1). Although other factors like lifestyle, pre-existing ailments (2, 7), environmental factors, genetics, and infectious agents (8) have equally been linked to different cancers, this study focuses on the few factors listed above.

Although the prevalence of smoking is somewhat greater among men than women (9), women may also have a higher risk of smoking-related cancers as seen in a recent study on lung cancer conducted for South African women (10). Even secondhand smoke can cause many of the same health problems as directly inhaling from cigarettes (11). Many studies conducted on African women have shown that any amount of alcohol can increase the risk of cancer (12–14). The negative effects of other factors like obesity (15) and breastfeeding rates (16) on the health of African women are becoming more prevalent in many ways, putting them at higher risk for multiple cancers. Different sources confirm that there are strong links between air pollution and carcinogenesis (17–19). A very recent article by Ke-Cheng Chen and colleagues provides biological evidence of the relationship between indoor air pollution and cancers in women. Their work found that cooking and burning incense are the leading sources of indoor air pollution with the strongest links to lung cancer especially among women in low- and middle-income countries (20, 21).

The bane of poor healthcare systems and policies in the African continent is a cause for real concern, especially concerning life-threatening noncommunicable diseases (NCDs) like cancer (2, 7). This concern is even more troubling when it is accompanied by a sparsity of quality far-reaching research to support policy development and external support (22–24). If the goal of African governments to reduce mortality from noncommunicable diseases by 30% by 2030 (2) is to be achieved, a critical look into cancer mortality on the continent would be a good first step. While there are some excellent specific research works on cancer of different types and for different African countries as seen in the review above, this work stands out for its novelty in method, scope, and overall value. The authors find that no recent study critically assesses cancer in Africa from a quantitative and statistical perspective, particularly about issues like survivorship. This research aims to provide concrete insight into the dynamics of cancer mortality, incidence, and survivorship among African women between 2016 and 2020. Due to time and data availability constraints, this study does not focus on a specific cancer type which might be a limitation in critically assessing the heterogeneity in the links between the studied factors and different cancer types. The study also assesses the chosen years since data collection began in 2020 before the pandemic. However, the authors are confident that this work provides a useful and recent tool for policy-making for the continent and a first step in understanding these links on a broader level. To achieve this aim, the research aims to answer the following groups of questions:

(a). Cancer Mortality and Incidence:
   (i) What are the levels of cancer incidence and mortality among African women in 2020?
   (ii) What is the mortality-to-incidence ratio of African women from cancer in 2020?
(b). Five-Year Survival and Relative Survival:
   (i) What is the average five-year survival rate of African women with cancer between 2016 and 2020?
   (ii) What is the average relative survival rate of African women with cancer between 2016 and 2020?

This work considers 53 of the African countries with sufficient verifiable data on Alcohol use, Breastfeeding prevalence, Smoking prevalence, and Obesity. The work also includes those with sufficient and verifiable data on outdoor and indoor air pollution, cancer mortality, cancer incidence, and cancer prevalence for each female age group from 0 to 85+ years. The study only uses data within the specified period of 2016–2020.

## 2 Methods

This research is conducted in two parts: Mortality-to-incidence ratio (MIR) and Relative Survival (RS) Rate in line with the two objectives of this work. The data collection is divided into two stages: data collection for MIR and data collection for relative survival rate.

### 2.1 Data collection

The final data for the first stage of this research as seen in the Supplementary Material has Mortality and Incidence as the response variables, and six predictor variables, including Alcohol prevalence, Smoking prevalence, Obesity prevalence, Breastfeeding, Outdoor Air Pollution, and Indoor Air Pollution. These were obtained after cleaning and merging relevant data from two primary sources: World Health Organization's Global Health Observatory (GLOBOCAN) (25) and the Cancer Atlas (26).

The final data for the second stage as seen in the Supplementary Material has five variables:

1. **Incidence:** An estimate of the sex- and age-specific incidence rates of cancer in a specific country (27). Having obtained the 2020 incidence estimates from (27), this study attempts to extrapolate for 2016–2019 estimates as follows:
   (a). Determine the proportion of females of each age cohort with cancer for 2020 relative to the total female population of the same age cohort in 2020. This is also referred to as the age-specific rate of incidence.
   (b). Multiply the 2020 age-specific rate of all age cohorts with the female population of the age cohorts in the year of interest. i.e., 2020 age-specific rate * 2016 0–4 years female population = 2016 0–4 years cancer incidence.
   (c). Where the results of (b) deviate from any observed trends as found in verified literature on the total cancer incidence and sex distribution of cancer incidence, the study attempts to simulate the computations by multiplying the results of (b) by small fractions until the results match the trends. All sources can be found in the Supplementary Data Sheet of this work.

**Note:** All population data used in this study are based on United Nations medium variant estimates (28, 29) obtained from PopulationPyramid.net (30).

2. **Mortality:** The age-specific mortality values are obtained similarly to the incidence described above.
3. **Mortality-to-incidence Ratio:** After obtaining the mortality and incidence values as explained above, they are converted to age-standardized rates. The age-standardized rate of mortality (ASMR) and age-standardized rate of incidence (ASIR) represent the respective age-specific results obtained above measured per 100,000 women in 2020 based on population data from the UN (30). The mortality-to-incidence ratio (MIR) is calculated by dividing the age-standardized mortality rate by the age-standardized incidence rate, allowing for a prompt international comparison of survival across countries (31).
4. **Cancer Prevalence:** The 5-year cancer prevalence estimates for 2020 include sex- and age-specific ratios of prevalence in a country (27).
5. **Cancer Survival Rate:** The cancer survival rate estimates for 2016–2020 were computed by dividing the prevalence estimate for each age group by the corresponding incidence estimate for the age group. i.e.,
  $$\frac{\mathrm{Cancer}\;\mathrm{Prevalence}\;\mathrm{of}\;\mathrm{females}\;0-4\;\mathrm{years}}{\mathrm{Cancer}\;\mathrm{Incidence}\;\mathrm{of}\;\mathrm{females}\;0-4\;\mathrm{years}}$$
6. **General Population Survival Rate:** The general population survival rate for each age group was obtained using life table data from the World Health Organization's Global Health Observatory data repository. The life table was obtained for each of the 53 countries used. After obtaining the respective country life table data, the survival rate was calculated for the different age groups based on already established methods in (32), found in (33) as follows:
  • Survival rate of youngest age cohort (0–5): $\frac{L_{0-1}+L_{1-5}}{\left(100000*5\right)}$
  • Survival rate of age cohorts from 5 to 84: $\frac{L_{x+5}}{L_{x}}$
  • Survival rate of the oldest age cohort (85+): $\frac{t_{80-85+}}{t_{75-80}}$

where:

Lx: The total number of person-years in the stationary population for each age interval. It can be viewed as the average population size between birthdays, taking into account the distribution of deaths throughout the year.

Tx: This column records the stationary population in the indicated age and subsequent intervals. It can be viewed as the total number of person-years that would be lived for a particular age cohort if the cohort were to progress through the remainder of the life table. The value of Tx represents the number of survivors in a particular age group and all older age groups.

x: The age group of interest.

x + 5: The next age group. If x = 0–4 then x + 5 = 5–9.

#### 2.1.1 Analysis of variance (ANOVA)

It compares the variance of the group means with the mean of all the data (numerator) and the variance of individual data points within each group (denominator). If the group means differ from one another (signal) more than the variation within groups (noise), then the F-ratio will exceed a critical value for significance (34). The null hypothesis in this study is that the mean values of all groups are the same. The alternative hypothesis is that not all the means are equal (some could be the same, but others are different). The test statistic is the F-ratio of the mean squares among all groups (MSA) to the error mean square (MSE): $F-\mathrm{ratio}\;=\frac{MS_{A}}{MS_{E}}$.

#### 2.1.2 Relative survival rate

The relative survival rate represents the ratio of two survival rates—the survival of the population of patients with cancer (which is the observed rate) divided by the expected survival of the general population. The expected survival rate is the probability of a population surviving from year to year specified by age, race, and sex. The relative rate attempts to estimate the effect of cancer alone on survival, excluding other death-causing factors.

### 2.2 Software used

This work uses Python programming language (Python 3) to perform data wrangling and all exploratory data analysis like mean, correlation, and display of visualizations. It also uses Python for the computation of the MIR. The Python code used is attached as a Supplementary File S1 to this work. All computations for the survival rates are done using Microsoft Excel 365. This work uses the Statistical Package for Social Sciences (SPSS) version 27 (35) to analyze variance.

## 3 Results

### 3.1 Data

Due to the size of the data sets used in this study, the tables of raw data and results are found in the Supplementary Information attached to this work.

### 3.2 Mortality, incidence, and MIR results

Table 1 above provides the full country-level results for mortality, incidence, and MIR among African women. The results show that Nigeria had the highest number of women with cancer deaths with 43 women deaths out of 100,000 Nigerian women, more than 6 times above the average mortality of 7 women deaths out of 100,000 African women. On the other extreme is Sao Tome and Principe with less than 1 woman death out of 100,000 women. The results for incidence resemble those of mortality closely. They show that Nigeria had the highest number of women per 100,000 women in the general population having reported cancer with 71 cases, more than 6 times above the average incidence of 12. On the other extreme is also Sao Tome and Principe with less than 1 case for the same population size (100,000 women). For the age-standardized mortality-to-incidence ratio, the results show that Mauritius had the lowest mortality-to-incidence rate for women (0.45) while Guinea had the highest (0.73).

**Table 1:** Mortality, incidence, and MIR results per country.

Country	Mortality	Incidence	Mortality (ASMR)	Incidence (ASIR)	MIR
Algeria	14,900	31,090	14	30	0.48
Angola	6,914	11,553	7	11	0.6
Benin	2,397	3,617	2	4	0.66
Botswana	618	1,171	1	1	0.53
Burkina Faso	5,447	7,740	5	8	0.7
Burundi	3,288	4,641	3	5	0.71
Cabo Verde	226	420	0	0	0.54
Cameroon	7,551	12,235	7	12	0.62
Central African Republic	1,158	1,612	1	2	0.72
Chad	3,545	5,142	3	5	0.69
Comoros	250	381	0	0	0.66
Congo	18,841	27,222	18	26	0.69
Ĉote d’Ivoire	6,540	9,896	6	10	0.66
Djibouti	314	479	0	0	0.66
Egypt	40,752	68,090	40	66	0.6
Equatorial Guinea	304	510	0	0	0.6
Eritrea	1,031	1,526	1	1	0.68
Eswatini	389	643	0	1	0.6
Ethiopia	32,970	50,598	32	49	0.65
Gabon	562	1,032	1	1	0.54
Gambia	415	575	0	1	0.72
Ghana	8,630	14,078	8	14	0.61
Guinea	3,698	5,069	4	5	0.73
Guinea-Bissau	518	720	1	1	0.72
Kenya	16,626	26,550	16	26	0.63
Lesotho	796	1,206	1	1	0.66
Liberia	1,507	2,121	1	2	0.71
Libya	2,171	3,913	2	4	0.55
Madagascar	8,026	12,447	8	12	0.64
Malawi	7,775	11,213	8	11	0.69
Mali	6,307	8,987	6	9	0.7
Mauritania	1,237	1,870	1	2	0.66
Mauritius	762	1,686	1	2	0.45
Morocco	14,940	30,199	14	29	0.49
Mozambique	10,966	15,406	11	15	0.71
Namibia	1,047	1,904	1	2	0.55
Niger	4,043	5,680	4	6	0.71
Nigeria	44,699	73,417	43	71	0.61
Republic of Congo	833	1,373	1	1	0.61
Rwanda	3,460	5,152	3	5	0.67
Sao Tome and Principe	48	70	0	0	0.69
Senegal	4,869	7,232	5	7	0.67
Sierra Leone	2,000	2,872	2	3	0.7
Somalia	4,612	6,411	4	6	0.72
South Africa	29,231	57,109	28	55	0.51
South Sudan	2,617	3,626	3	4	0.72
Sudan	9,707	16,529	9	16	0.59
Tanzania	16,147	25,165	16	24	0.64
Togo	1,860	2,889	2	3	0.64
Tunisia	4,576	8,973	4	9	0.51
Uganda	12,978	19,479	13	19	0.67
Zambia	4,881	7,863	5	8	0.62
Zimbabwe	6,730	10,339	7	10	0.65

**Figure 1:**
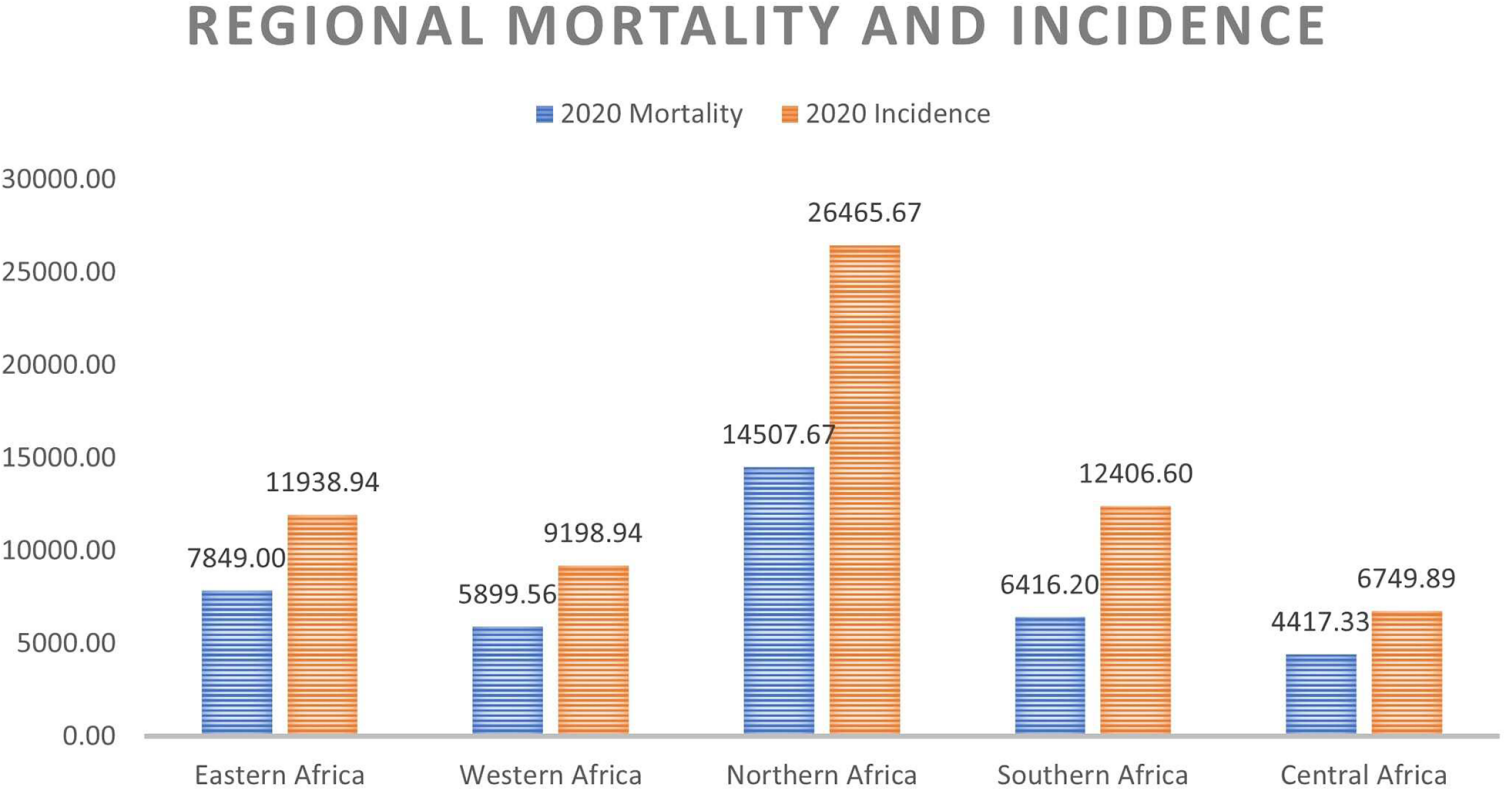
Average mortality and incidence for each region.

**Figure 2:**
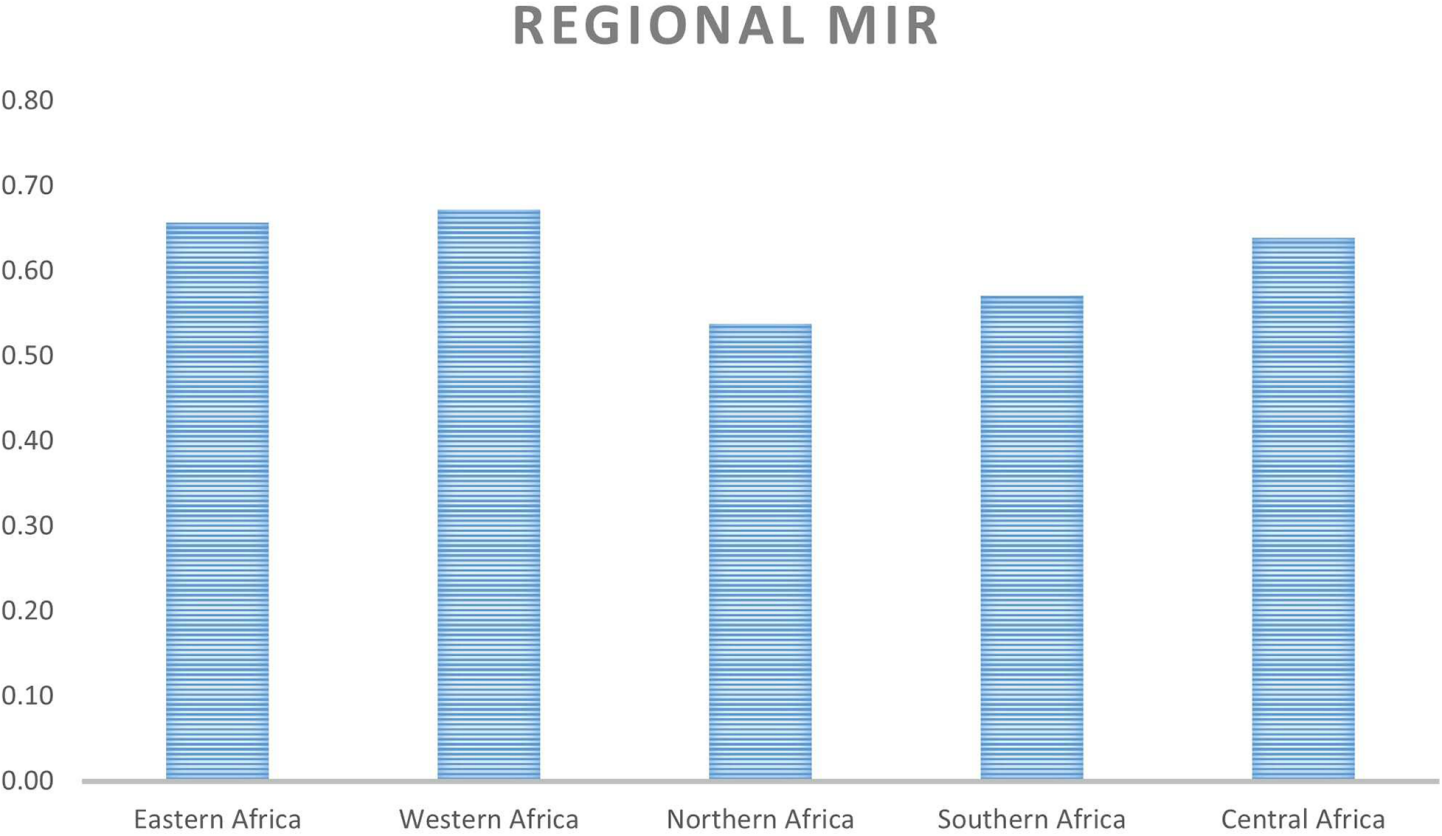
Average MIR for each region.

On a regional scale, Figures 1, 2 show that although Northern Africa has the highest cancer incidence and mortality among women in 2020, the region has the least mortality-to-incidence ratio for the same cohort. The trend appears similar for Southern Africa but does not repeat for other regions.

### 3.3 Correlation

**Figure 3:**
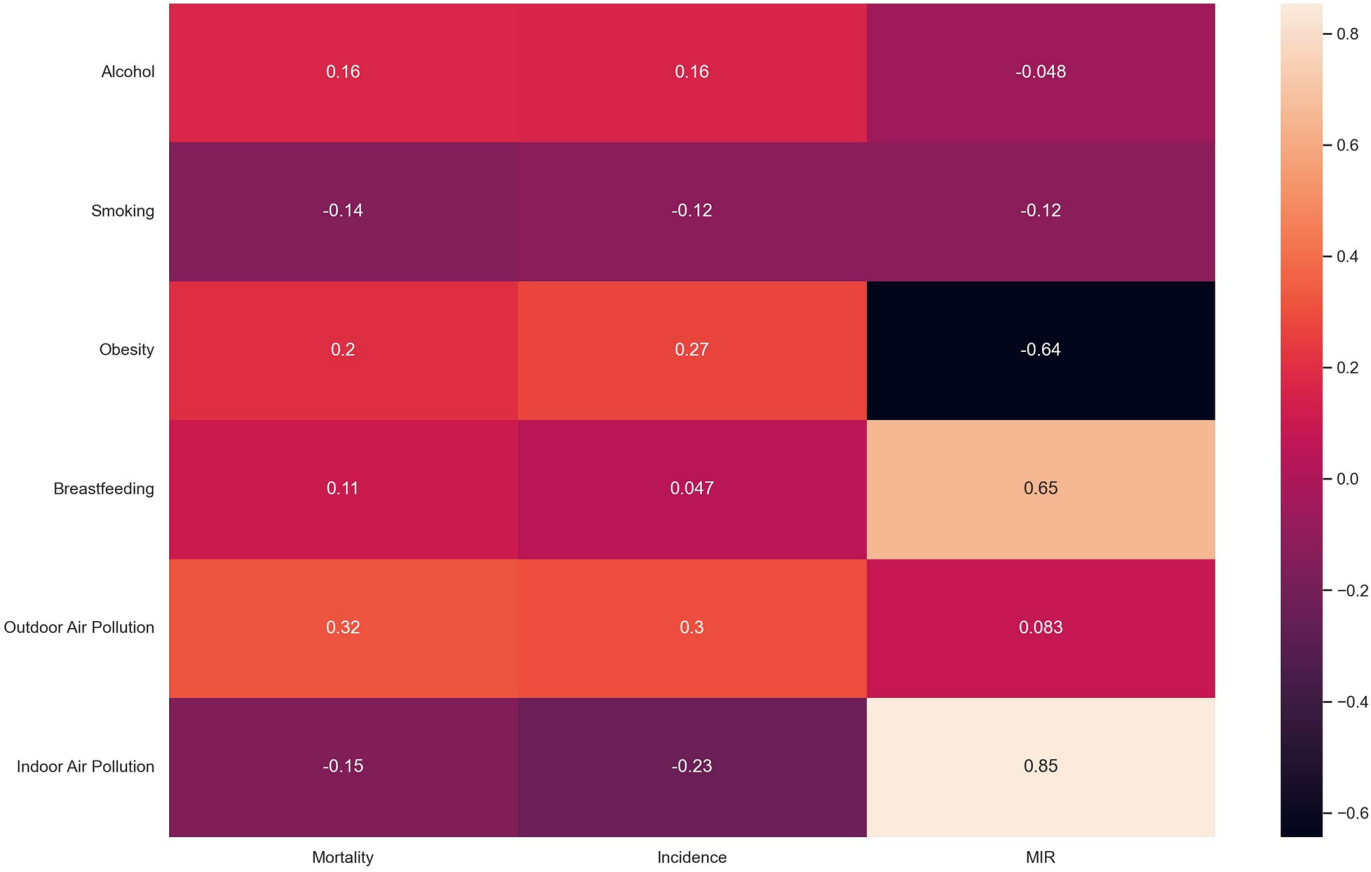
Correlation analysis.

The correlation analysis of all the variables for the 2020 data set is presented in the heatmap below:

The heatmap in Figure 3 above shows that alcohol, obesity, and breastfeeding have a slightly positive correlation with mortality while smoking and indoor air pollution have a slightly negative correlation with mortality. The correlation between outdoor air pollution is highest as this factor shows a fair positive correlation. For cancer incidence, the pattern is similar for all examined factors. However, for MIR, the pattern is very different. Here, a slightly negative correlation exists between alcohol use and smoking with MIR while a slightly positive relationship exists between MIR and outdoor air pollution. There is a substantial positive correlation of breastfeeding with the mortality-to-incidence ratio while there is a substantial negative correlation between obesity and MIR. On the other hand, the results show a nearly perfect correlation between indoor air pollution and MIR.

### 3.4 Survival rates

The general survival rate, cancer survival rate, and relative survival rate for women in the different countries are summarized in the table below according to their regions.

#### 3.4.1 Summary table

Table 2 shows the different regions’ average survival rates (for female cancer patients, the general female population, and relative survival). The table shows that women in Northern and Southern Africa have a higher chance of surviving cancer than women in other sub-regions. The results of the relative survival rate for the different regions also show that even when all other death-causing factors are eliminated, Northern and Southern Africa still have higher cancer survival rates than other regions.

**Table 2:** Mean survival rates for Africa, 2020.

Region	Cancer survival rate	General survival rate	Relative survival rate
Eastern Africa	39%	92%	42%
Western Africa	40%	92%	43%
Northern Africa	53%	95%	56%
Southern Africa	51%	91%	56%
Central Africa	44%	91%	48%

Supplementary Table S7 shows that in Eastern Africa, women from Eritrea have the lowest survival rate and relative survival rate. Women from Mauritius have the highest survival rate and relative survival rate in this region. This is also in line with the MIR previously discussed in this work where Mauritius had the lowest mortality-to-incidence ratio in the whole of Africa. It is also interesting to observe that although Somalia had a lower cancer survival rate than South Sudan, due to its corresponding low general survival rate, it appears to have a better relative survival rate than South Sudan.

Supplementary Table S7 shows that in Western Africa, women from Niger have the lowest survival rate and relative survival rate while women from Cabo Verde have the highest cancer survival rates and relative survival rate. Supplementary Table S7 shows that in Northern Africa, women from Sudan have the lowest survival rate and relative survival rate while women from Tunisia have the highest cancer survival rates and relative survival rate.

Supplementary Table S7 shows that women from Lesotho and Eswatini have the lowest survival rate in Southern Africa. However, due to Lesotho's already low general survival rate, the women have a slightly higher relative survival rate than Eswatini. Women from Namibia have by a wide margin the highest cancer survival rates and relative survival rates. Far higher than women from wealthier South Africa within the same region. Supplementary Table S7 shows that in Central Africa, women from the Central Africa Republic have the lowest survival rate and relative survival rate. Women from Gabon have the highest cancer survival rates and relative survival rates.

**Figure 4:**
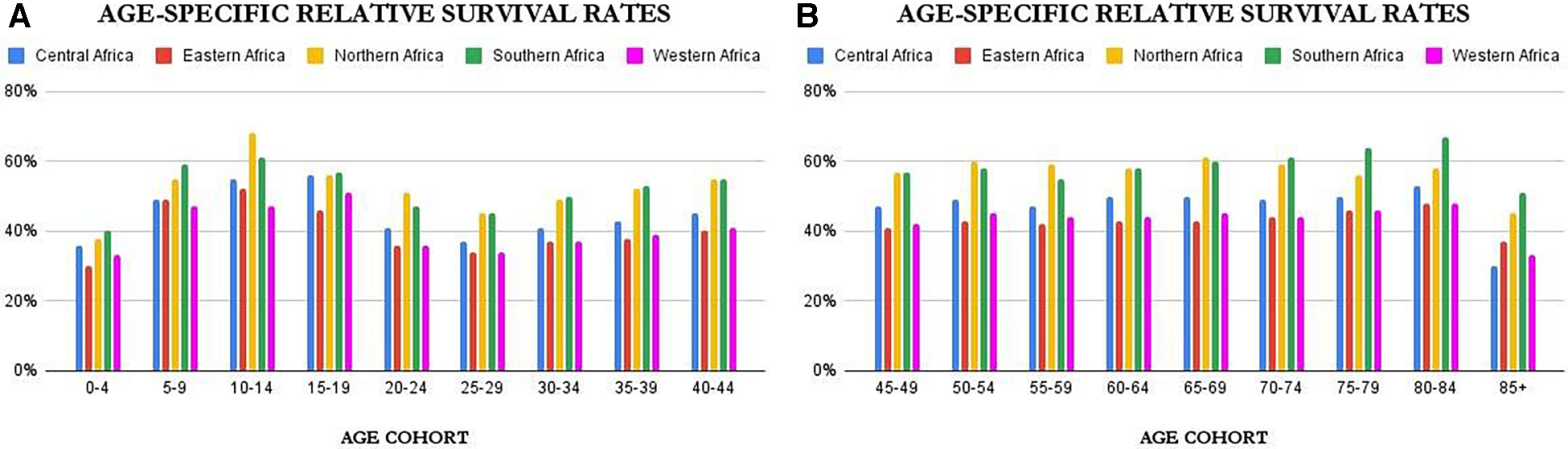
Age-Specific relative survival rate.

Age-specific survival rates can be found in Supplementary Tables S8–S13 of this work. However as a summary, Figures 4A,B above provide an overview of the age-controlled results of relative survival for the different countries. From the figures, it is clear that relative survival is highest among females aged 5–19 and 80–84. The lowest relative survival rates are seen for infants (0–4), adolescents and young adults (25–29), and the very elderly (85+). The figures show that for most of the age cohorts, the trend is the same with Northern and Southern Africa dominating, except in females 15–20 years where Central Africa comes very close to Northern and Southern Africa.

### 3.5 Analysis of variance

The following results are outputs from the SPSS analysis of the full data including the relative survival rate for females in each age group in each region. Tests of Homogeneity of Variances and *Post Hoc* test results are presented in the Supplementary Information (SI) of this work.

#### 3.5.1 Tests of homogeneity of variances

Using the Levene statistic based on the medians as seen in Supplementary Table S15, we have a value of 0.153. Since this value is greater than 0.05, the result is NOT significant and we conclude that the requirement for homogeneity of variance has been met. Therefore, the study can trust the results of the ANOVA test presented in Table 3 below.

**Table 3:** ANOVA table showing the statistical significance of any differences between the different countries’ mean survival rate (Africa, 2020).

	Sum of squares	df	Mean square	F Sig.
Between groups	0.322	4	0.080	19.648 0.00
Within groups	0.348	85	0.004	
Total	0.669	89		

#### 3.5.2 ANOVA

The ANOVA result in Table 3 is clearly significant. This is because we have an F value of 19.648 with a *p*-value of 0.00 which is less than 0.05. This means there is a statistically significant difference between the means of survival rates in the different regions.

#### 3.5.3 *Post Hoc* tests: tukey HSD

The results show that there is a significant difference, the study proceeds to find out which pairs of means contribute to this result. For this, the study refers to Supplementary Table S16 which clearly shows the groups reaching significance are the mean differences between Western Africa against the Northern and Southern African groups; Central Africa against the Northern and Southern African groups; and Eastern Africa against the Northern and Southern African groups.

## 4 Discussion

### 4.1 Descriptive statistics

**Figure 5:**
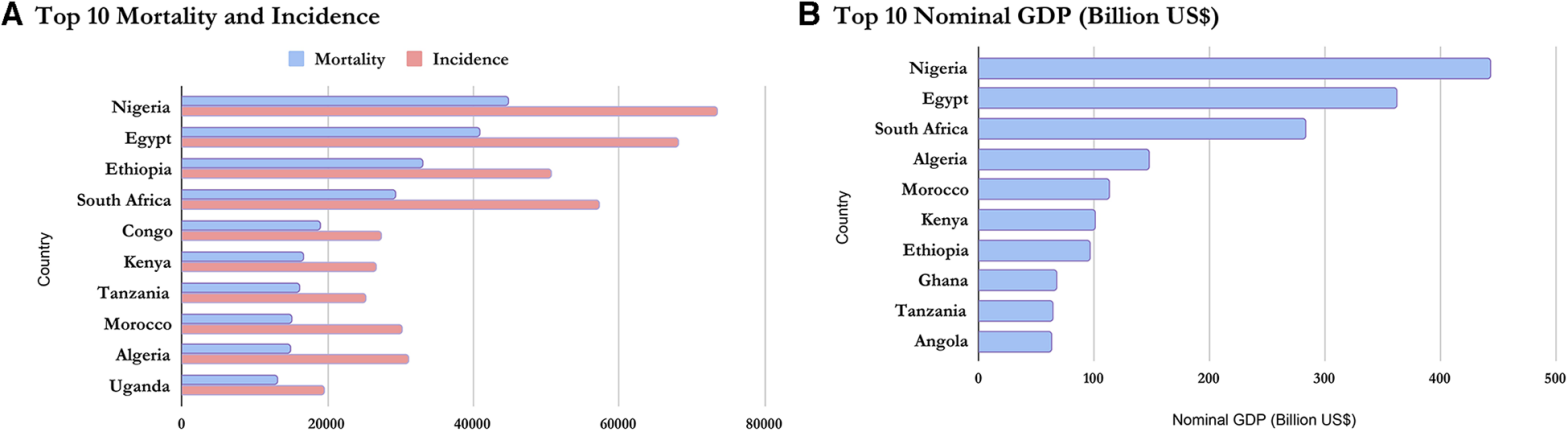
Comparing the top 10 economies in Africa with the top mortality and incidence.

**Figure 6:**
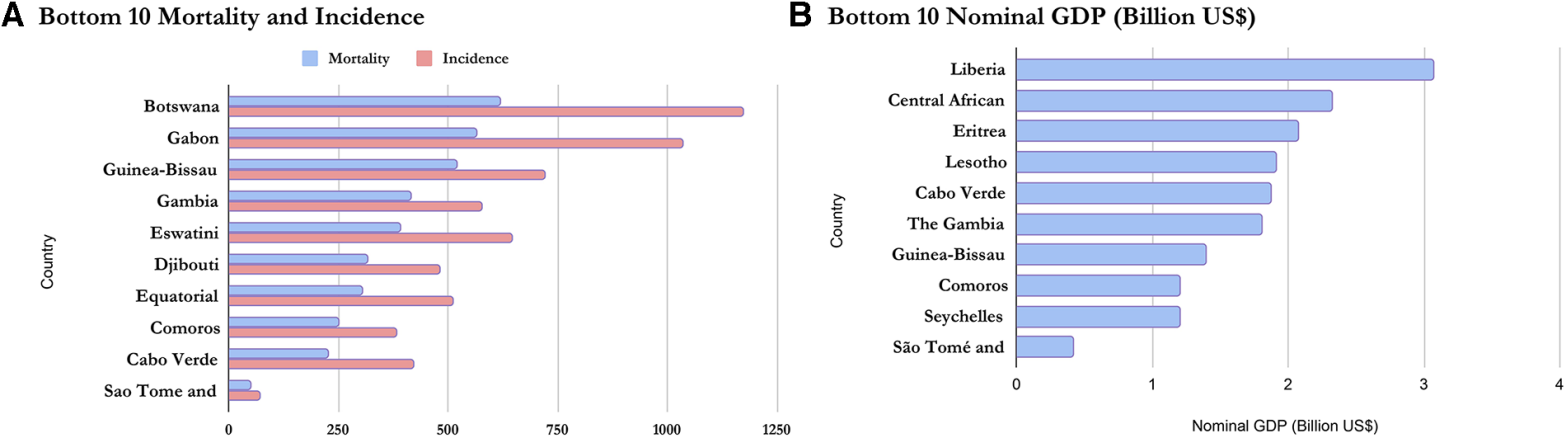
Comparing the bottom 10 economies in Africa with the bottom mortality and incidence.

Based on the Nominal GDP for 2020 obtained from the International Monetary Fund World Economic Outlook Database (36), it can be inferred that mortality and incidence may be related to GDP as wealthier countries seem to appear at the top of the list while poorer countries have lower incidence and mortality. This is shown in Figures 5, 6 and confirms the earlier assumptions and results of previous works (37) on the topic.

### 4.2 Correlation

The heatmap in Figure 3 above shows a weak positive relationship between alcohol, obesity, and outdoor air pollution with cancer incidence and mortality in African women. This is also in line with previous research as stated earlier in this report. The relationship between breastfeeding and cancer incidence (*r* = 0.047) is also in line with existing studies (38) which also show no real relationship between breastfeeding patterns and cancer prevention or incidence. The heatmap showed a weak negative relationship between indoor air pollution with cancer incidence and mortality in African women. This is contrary to the initial hypothesis based on existing research by Chen and colleagues for Chinese households (20) hence, further studies may be required to lend more credence to this argument. The results may also show this pattern because, unlike Chen's work which focused on lung cancers, this work takes all cancers as a whole. The heatmap also showed a weak negative correlation between smoking and cancer incidence and mortality when a previous study showed the reverse (9). However, it may also be due to the misclassification of self-reported smoking as explained by Pakzad et al. which proved that after adjusting for confounders, the correlation between smoking and breast cancer changed from negative to positive (11). It may also be because this work takes all cancers as a whole.

### 4.3 Survival rate

Figure 2 and Table 2 show the average survival rates for the different regions. The table shows that women in Northern and Southern Africa have a higher chance of surviving cancer than women in other sub-regions. This result is in line with existing research on the region which shows that due to the high prevalence of hypertension among the population, NCDs mortality is high (4). The figure also shows the same result despite using a different metric. This is similar to the results from a recent study (8) showing that there are disparities in cancer incidence and fatality rates across the different African regions (on average and for most cancer types). In this study by Hamdi and colleagues, the authors suggest that these patterns may be due to increased urbanization, tobacco and alcohol consumption, and the adoption of *Western* habits. Early detection and cancer screening are possible reasons for the high incidence rates reported in these regions against other regions with much lower reported numbers. The under-diagnosis of the disease in Eastern, Western, and Central Africa may be one of the reasons for the high fatality and low survivorship in those regions due to the lack of therapy at the early stages.

Concerning age-specific results for relative survival, the results show low relative survival rates for infants (0–4), young adults (20–29), and the very elderly (85+). Apart from the fact that infant mortality in Africa is still generally high (39), low relative survival rates for the age group 0–4 might be due to the inability to obtain an accurate diagnosis, death from side effects of treatments, or other healthcare-related issues (40). Low relative survival rates for the age group 20–29 might be because women in this cohort have higher lifestyle transitions coupled with poor awareness and lower disposable income or savings (41). The potential reasons for this could be delays in the diagnosis of certain cancers arising from a combination of factors such as higher rates of women lacking health insurance coverage, limited availability of cost-effective early detection methods, and the relatively infrequent occurrence and sporadic nature of cancer within this particular age group (42–44). The 85+ age group has a low general survival rate (as seen in Supplementary Table S9) which may be due to a weakened immune system. This trend is similar in the UK and Brazil (45, 46). These results are, however, a general overview as cancer survivorship might differ among cancer types.

### 4.4 Analysis of variance

The results of the Analysis of Variance confirm that the differences highlighted above between Northern or Southern Africa and the other African regions are statistically significant. This suggests that the variation in survival rates observed among the regions is unlikely to be due to random chance and may have meaningful underlying factors contributing to the differences.

### 4.5 Limitations

Despite meeting its set objectives, the results presented are limited to the countries within the scope and inclusion criteria leading to the exclusion of France, La Reunion` due to the sparsity of verifiable data for the country. The study observes the difficulty in obtaining structured and up-to-date data on the different variables used. The results are therefore also limited by the authenticity of the different sources of data used. In computing the survival rates from the prevalence data, the authors found some data points that showed unusual patterns across all countries for a single age cohort. The authors assumed this was a data entry error from the source. To handle this assumed error, the authors used the difference between the given value and the value before it, the result of which seemed to fit perfectly for most countries and helped align the data to the observed pattern.

## 5 Conclusion

The study successfully provided concrete insight into the dynamics of cancer incidence and survivorship among women in different African regions between 2016 and 2020. To achieve this aim, the work fulfilled two key objectives related to cancer incidence (and mortality-to-incidence ratio) and relative survival rates from cancer. In fulfilling the first objective, the work shows that although the bad health habits of women in richer countries need to be highlighted to tame cancer incidence, poorer countries may need more support for healthcare and effective awareness to increase detection and their chances of surviving cancer. That might be inferred from both the lower incidence and poorer survival in women from poorer countries that never detect cancers or only detect them within a year of survival. In summary, the result stresses that different areas of focus should apply to different classes of countries based on their wealth. This conclusion shows that for cancer mortality, the GDP may be a major confounder, aligning with previous studies that show that the chances of identifying cancer, and how early or late, may well depend on the level of health care and hence the GDP.

In fulfilling the second objective, however, a different relationship is observed between GDP and relative survival rates as seen in the Supplementary Information. In this case, there was no smooth distinction between rich and poor. This implies that the GDP does not strongly influence prevalence as it does on mortality because relative survival rates are tied to prevalence. This research has not been able to find concrete reasons for this outcome and why some top economies like Nigeria had low relative survival rates as compared to smaller countries like Mauritius for instance, but not as low as a country like Eritrea. The study also observed that the countries on the top (North) and bottom (South) of the continent showed better relative survival rates. Further work is, therefore, recommended to try to understand this unique finding as this work exposes possible links between climate regions and survivorship dynamics [like recent work by Hamdi and colleagues (8)]. Future research can attempt to investigate the results for specific cancer types to provide more insights on a deeper more focused level and specific research for each age cohort as cancer incidence and mortality has been shown to vary with age.

Concerning cancer research, this study recommends that the healthcare systems in Africa be treated with top priority, especially concerning funding, record keeping, and intervention. It, therefore, recommends that as part of efforts to control cancer incidence and increase survivorship, modern technologies should be employed for accurate data collection, storage, processing, and analysis. Judging by the trends highlighted in the introduction of this work, urgent action is needed to slow down the rate of NCDs. Based on the correlation findings between the studied risk factors and cancer, implementing certain measures can be beneficial. These include raising tobacco taxes, placing limitations on alcohol advertisements, adjusting the composition of food products to contain less salt, sugar, and fat, providing vaccinations for cervical cancer to girls, and effectively managing hypertension and diabetes (4). Moreso, adopting positive lifestyle habits, overall vaccination against primary ailments, and providing access to genetic testing can help boost cancer care (8).

Despite answering the questions it set out to, the work has produced more questions for future research than it had in the beginning. (i) Could the difference in the results of the first part (MIR) from the second part (Relative survival) be because the prevalence numbers are purely based on detected cases while mortality includes a huge percentage of undetected cases, especially in poorer countries? (ii) Could this then be the ideal situation for cancer survival if detection was more in poorer countries? (iii) Maybe the more healthy habits and lower exposure to pollution of poorer countries help them live longer even with cancer?

## Data Availability

The original contributions presented in the study are included in the article/Supplementary Material, further inquiries can be directed to the corresponding author.
