# Supplementary material for: Cancer incidence, mortality, and survivorship in African women: a comparative analysis (2016–2020)
Source: Front Glob Womens Health. 2024 Jan 11;4:1173244. doi: 10.3389/fgwh.2023.1173244 (PMC10808777; doi:10.3389/fgwh.2023.1173244)
Supplement: Supplementary file 1 [file Datasheet1.pdf]

SUPPLEMENTARY INFORMATION AND DATA FOR "CANCER INCIDENCE, MORTALITY, AND SURVIVORSHIP IN AFRICAN WOMEN: A COMPARATIVE ANALYSIS (2016-2020)"

This supplementary material provides more detailed data from the different sources outlined in the main work's Method section.

1 Full Data

1.1 Cancer Incidence by Type and Region, 2020

Table 1: Cancer incidence of different cancer types excluding non-melanoma skin cancers given for the African regions, 2020

CANCER TYPE	INCIDENCE				
	C/Africa	E/Africa	N/Africa	S/Africa	W/Africa
Breast	17896	45709	57128	16526	49339
Cervixuteri	15646	54560	6971	12333	27806
Colorectum	2722	9418	10196	3765	6037
Non-Hodgkinlymphoma	2280	6990	6154	1556	5644
Ovary	2279	7298	6496	1575	6615
Liver	1868	5101	11445	975	6011
Stomach	1614	5042	3983	754	3509
Leukaemia	1172	4756	4833	910	2626
Corpusuteri	1001	3550	3706	2342	3425
Oesophagus	793	7623	1504	1501	1091
Kidney	776	2677	1958	527	2159
Lung	767	3449	3869	2895	1726
Kaposisarcoma	729	5526	85	1757	721
Vulva	612	2025	747	487	1273
Lip,oralcavity	552	1963	1489	827	1281
Thyroid	535	4516	6833	1135	1599
Bladder	525	2874	3458	603	1740
Pancreas	509	1961	2630	1032	1699
Melanomaofskin	506	1321	490	917	618
Nasopharynx	447	1216	1126	72	653
Brain,centralnervoussystem	427	2037	4019	408	1707
Multiplemyeloma	421	1113	1057	550	813
Vagina	278	790	275	218	440
Salivaryglands	248	727	370	104	817
Hodgkinlymphoma	247	1162	1601	309	1110
Gallbladder	122	771	2095	274	369
Larynx	102	413	438	169	300
Oropharynx	76	143	169	120	241
Hypopharynx	42	203	279	55	133
Mesothelioma	13	41	193	93	14
TOTAL	55205	184975	145597	54789	131516

1.2 Cancer Mortality by Type and Region, 2020

Table 2: Cancer mortality of different cancer types excluding non-melanoma skin cancers given for the African regions, 2020

CANCER TYPE	MORTALITY				
	C/Africa	E/Africa	N/Africa	S/Africa	W/Africa
Breast	9500	24047	21524	5090	25626
Cervixuteri	10572	36497	4033	6867	18776
Colorectum	2006	6871	5630	1891	4431
Non-Hodgkinlymphoma	1466	4631	3445	797	3678
Ovary	1675	5258	4294	1047	4734
Liver	1760	4824	10944	873	5803
Stomach	1437	4454	3238	621	3096
Leukaemia	912	3556	3482	680	1999
Corpusuteri	329	1152	790	645	1126
Oesophagus	755	7160	1458	1400	1038
Kidney	494	1777	1051	229	1451
Lung	713	3147	3433	2531	1587
Kaposisarcoma	416	3263	40	445	430
Vulva	367	1176	357	221	737
Lip,oral cavity	352	1264	727	318	821
Thyroid	218	1334	1055	118	612
Bladder	301	1628	2018	287	1011
Pancreas	499	1916	2538	999	1661
Melanomaofskin	214	551	224	229	268
Nasopharynx	316	843	669	50	444
Brain,centralnervoussystem	357	1720	3292	334	1396
Multiplemyeloma	363	934	895	430	691
Vagina	162	473	125	74	268
Salivaryglands	160	474	157	43	521
Hodgkinlymphoma	117	523	488	67	510
Gallbladder	109	680	1507	213	330
Larynx	70	283	295	108	205
Oropharynx	51	93	72	57	163
Hypopharynx	34	159	152	24	105
Mesothelioma	13	40	180	89	14
TOTAL	35738	120728	78113	26777	83532

1.3 Cancer Prevalence by Type and Region, 2020

Table 3: 5-year cancer prevalence of different cancer types excluding non-melanoma skin cancers given for the African regions, 2020

CANCER TYPE	FIVE-YEAR PREVALENCE				
	C/Africa	E/Africa	N/Africa	S/Africa	W/Africa
Breast	33444	88696	156965	50442	99673
Cervixuteri	28049	98660	16631	29987	50230
Colorectum	4684	16568	24210	9058	10520
Non-Hodgkinlymphoma	4503	13864	15939	4315	11590
Ovary	4087	13269	15548	3795	12241
Liver	2675	7181	12232	1137	7558
Stomach	2275	7270	5652	1051	5082
Leukaemia	2373	9773	12756	2380	5438
Corpusuteri	2066	7668	10744	7092	7325
Oesophagus	863	8565	1651	1619	1206
Kidney	2335	7130	5565	1516	4899
Lung	953	4404	4826	3532	2179
Kaposisarcoma	1613	11341	215	4884	1476
Vulva	1279	4351	1882	1438	2819
Lip,oralcavity	1093	3867	3586	2090	2437
Thyroid	1154	9548	20725	3537	3592
Bladder	899	4937	8406	1457	2915
Pancreas	516	1925	2391	858	1601
Melanomaofskin	982	2668	1287	2493	1197
Nasopharynx	843	2347	3053	187	1307
Brain,centralnervoussystem	851	4175	10947	1103	3474
Multiplemyeloma	688	1944	2372	1259	1399
Vagina	512	1394	636	534	784
Salivaryglands	594	1594	1059	316	1881
Hodgkinlymphoma	515	2648	4947	1078	2783
Gallbladder	143	967	2463	295	475
Larynx	180	790	1141	449	572
Oropharynx	128	243	381	282	402
Hypopharynx	55	247	453	97	147
Mesothelioma	17	50	239	113	22
TOTAL	100369	338084	348902	138394	247224

The above data are pulled from the WHO Global Cancer Observatory [1] and contain region-specific data. The data presented excludes non-melanoma skin cancer and is as obtained from the data source. However, the country-specific data are not presented in this work due to their size but can be obtained from the same source [1].

2 Exploratory Data Analysis

Table 4: 2020 Median Values for all Variables. The following table of actual values for the respective variables is presented as obtained from the different sources as described in the main paper

Country	Mortality	Incidence	Alcohol	Smoking	Obesity	Breastfeeding	OAP	IAP
Algeria	14900	31090	0.2	2.2	34.9	55.1	39	0.32
Angola	6914	11553	2.4	1.6	12.1	92.6	32	56
Benin	2397	3617	1.1	1	14.2	85.6	39	90
Botswana	618	1171	2.1	3.5	29.3	44.5	23	46
Burkina Faso	5447	7740	2.4	3.5	8.1	97.2	43	95
Burundi	3288	4641	1.9	0.9	8.6	96.4	39	99
Cabo Verde	226	420	2.5	2.6	16.3	83.1	35	39
Cameroon	7551	12235	2.3	0.5	16.4	85.5	73	65
Central African Republic	1158	1612	1.4	1.4	10.9	90.9	57	98
Chad	3545	5142	1.6	1.9	8.9	90.4	66	96
Comoros	250	381	0.1	4.3	12.2	79.4	21	76
Congo	18841	27222	0.9	0.9	9.7	95.6	45	96
Côte d'Ivoire	6540	9896	2.5	1.4	15.2	92.1	26	81
Djibouti	314	479	0.6	2.8	18.3	55	46	27
Egypt	40752	68090	0.3	0.1	41.1	85.5	87	0.8
Equatorial Guinea	304	510	2.5	1.2	12.6	60.4	53	79
Eritrea	1031	1526	0.8	0.6	7.6	96.3	48	80
Eswatini	389	643	1.1	0.8	26.2	66.5	17	37
Ethiopia	32970	50598	0.8	0.2	6.9	97.3	39	89

Gabon	562	1032	2.9	2.2	20.3	60.5	44	13
Gambia	415	575	2	0.8	14.8	98.7	34	95
Ghana	8630	14078	1.4	0.3	16.6	95.2	35	73
Guinea	3698	5069	1	1.4	11.5	94.9	26	98
Guinea-Bissau	518	720	1.7	1	13.7	97.8	30	97
Kenya	16626	26550	1.4	1	11.1	89.4	29	80
Lesotho	796	1206	1.5	0.3	26.7	79.8	28	59
Liberia	1507	2121	2.4	1.7	14.2	95.2	18	97
Libya	2171	3913	0	0.4	39.6	73.95	54	0.083
Madagascar	8026	12447	0.5	1.5	7.5	92.5	23	99
Malawi	7775	11213	0.7	3	9.1	98.3	24	95
Mali	6307	8987	0.3	2.3	12.4	94.1	39	97
Mauritania	1237	1870	0.1	2.9	18.5	89.5	47	53
Mauritius	762	1686	1.6	1.5	15.7	73.95	14	2.5
Morocco	14940	30199	0.2	0.8	32.2	66	33	1.5
Mozambique	10966	15406	0.4	3.6	10.5	93	21	95
Namibia	1047	1904	1.8	7.8	25.4	72.1	25	54
Niger	4043	5680	0.3	0.1	8.7	95.8	94	98
Nigeria	44699	73417	5.1	0.8	13.1	88.7	72	72
Republic of Congo	833	1373	2.2	1.1	13.5	86.2	47	63
Rwanda	3460	5152	2.8	3.8	9.3	95.7	43	99
Sao Tome and Principe	48	70	1.4	1	16.9	95.8	29	60
Senegal	4869	7232	0.6	0.3	12.9	99.4	41	84
Sierra Leone	2000	2872	2.5	7.9	13.3	87.8	22	97
Somalia	4612	6411	0.1	1.6	12.3	61	32	99
South Africa	29231	57109	2.8	5.5	39.6	73.7	25	11
South Sudan	2617	3626	1.5	1.7	22.75	87.4	46	99

Sudan	9707	16529	0.2	0.4	22.75	91.5	55	60
Tanzania	16147	25165	2.3	2.2	12.7	95.8	29	96
Togo	1860	2889	1.2	1.1	12.5	96.2	36	93
Tunisia	4576	8973	0.2	3	34.3	53.7	38	0.27
Uganda	12978	19479	3.1	1.3	8.6	91.8	50	97
Zambia	4881	7863	0.8	2.5	12.4	94.7	27	85
Zimbabwe	6730	10339	0.6	1.3	25.3	89	22	67

2.1 Summary of Exploratory Data

Table 5: 2020 Median Values for all Variables. To give a general overview of the initial data for 2020 collected from the WHO GCO, the following regional table of median values is presented

Region	Mortality	Incidence	Alcohol	Smoking	Obesity	Breastfeeding	OAP	IAP
East	4881	7863	0.8	1.6	11.1	92.5	29	95
West	3048	4343	1.55	1.25	13.5	95.05	35.5	94
North	12304	23364	0.2	0.6	34.6	69.98	46.50	0.56
South	796	1206	1.80	3.50	26.70	72.10	25.00	46.00
Central	1158	1612	2.20	1.20	12.60	90.40	47.00	65.00

Table 6: The following table shows the median Mortality-to-Incidence Ratio per Region

REGION	MIR
Eastern Africa	0.656168
Western Africa	0.684818
Northern Africa	0.532396
Southern Africa	0.549895
Central Africa	0.617164

3 Survival Rates

3.1 Summary of Country Survival Rates

Table 7: The following table shows the country-specific survival rates and includes the cancer survival rate, general survival rate, and relative survival rate

COUNTRY	Cancer Survival rate	General survival rate	Relative survival rate
Algeria	59%	96%	62%
Angola	51%	92%	56%
Benin	37%	92%	40%
Botswana	58%	91%	63%
Burkina Faso	33%	92%	36%
Burundi	33%	91%	36%
Cabo Verde	55%	95%	58%
Cameroon	49%	92%	53%
Central African Republic	29%	88%	34%
Chad	33%	91%	36%
Comoros	46%	93%	49%
Congo	35%	91%	39%
Côte d'Ivoire	44%	92%	48%
Djibouti	38%	92%	41%
Egypt	48%	94%	51%
Equatorial Guinea	50%	92%	55%
Eritrea	25%	91%	28%
Eswatini	39%	91%	43%
Ethiopia	38%	93%	41%
Gabon	56%	93%	60%
Gambia	48%	92%	52%
Ghana	43%	93%	47%
Guinea	38%	91%	42%
Guinea-Bissau	36%	90%	40%
Kenya	38%	92%	41%

Lesotho	39%	87%	44%
Liberia	40%	92%	44%
Libya	60%	96%	63%
Madagascar	42%	91%	46%
Malawi	33%	93%	36%
Mali	34%	91%	37%
Mauritania	42%	93%	45%
Mauritius	63%	95%	66%
Morocco	53%	94%	57%
Mozambique	35%	91%	39%
Namibia	69%	93%	75%
Niger	32%	91%	35%
Nigeria	42%	92%	45%
Republic of Congo	48%	92%	52%
Rwanda	39%	93%	42%
Sao Tome and Principe	43%	93%	46%
Senegal	39%	93%	42%
Sierra Leone	36%	89%	40%
Somalia	27%	87%	31%
South Africa	53%	93%	56%
South Sudan	28%	92%	30%
Sudan	39%	93%	42%
Tanzania	39%	93%	42%
Togo	38%	92%	41%
Tunisia	62%	96%	65%
Uganda	39%	93%	42%
Zambia	46%	92%	50%
Zimbabwe	51%	90%	56%

3.2 Country Age-specific General Survival Rates (0-44 years)

Table 8: The table below contains Country Age-specific General Survival Rates for females aged 0-44years as obtained from the WHO Global Health Observatory (Africa, 2020) [2]

COUNTRY	0-4	5-9	10-14	15-19	20-24	25-29	30-34	35-39	40-44
Algeria	98%	100%	100%	100%	100%	100%	100%	100%	99%
Angola	95%	98%	99%	99%	99%	99%	98%	98%	97%
Benin	93%	97%	99%	99%	99%	99%	99%	99%	98%
Botswana	97%	99%	100%	100%	99%	99%	98%	97%	96%
Burkina Faso	94%	97%	99%	99%	99%	99%	99%	99%	98%
Burundi	96%	98%	99%	99%	99%	99%	99%	98%	98%
Cabo Verde	99%	100%	100%	100%	100%	100%	100%	100%	99%
Cameroon	95%	98%	99%	99%	99%	99%	98%	98%	97%
Central African Republic	91%	97%	99%	99%	99%	98%	97%	96%	95%
Chad	92%	96%	99%	99%	99%	99%	99%	98%	98%
Comoros	95%	99%	100%	100%	100%	99%	99%	99%	99%
Congo	96%	99%	100%	100%	99%	99%	99%	98%	97%
Côte d'Ivoire	94%	98%	99%	99%	99%	99%	99%	98%	98%
Djibouti	95%	99%	99%	100%	99%	99%	99%	98%	98%
Egypt	98%	100%	100%	100%	100%	100%	100%	100%	99%
Equatorial Guinea	94%	98%	99%	99%	99%	99%	98%	97%	96%
Eritrea	97%	99%	100%	100%	99%	99%	99%	98%	98%
Eswatini	96%	99%	99%	99%	99%	98%	97%	96%	95%
Ethiopia	96%	99%	100%	100%	100%	99%	99%	99%	98%
Gabon	97%	99%	99%	100%	100%	99%	99%	98%	98%
Gambia	96%	99%	100%	100%	99%	99%	99%	98%	98%
Ghana	97%	99%	100%	100%	99%	99%	99%	98%	98%
Guinea	93%	97%	99%	99%	99%	99%	98%	98%	97%
Guinea-Bissau	94%	98%	99%	99%	99%	99%	98%	98%	97%

Kenya	97%	99%	100%	100%	99%	99%	99%	98%	97%
Lesotho	93%	98%	100%	99%	98%	97%	95%	93%	92%
Liberia	94%	98%	99%	99%	99%	99%	99%	98%	98%
Libya	99%	100%	100%	100%	100%	100%	100%	99%	99%
Madagascar	96%	99%	99%	99%	99%	99%	99%	98%	98%
Malawi	97%	99%	99%	100%	99%	99%	99%	98%	98%
Mali	93%	97%	99%	99%	99%	99%	99%	98%	98%
Mauritania	95%	98%	100%	100%	100%	99%	99%	99%	99%
Mauritius	99%	100%	100%	100%	100%	100%	100%	99%	99%
Morocco	98%	100%	100%	100%	100%	100%	100%	99%	99%
Mozambique	94%	98%	99%	99%	99%	98%	97%	96%	96%
Namibia	97%	99%	100%	100%	99%	99%	99%	98%	97%
Niger	94%	97%	99%	99%	99%	99%	99%	99%	98%
Nigeria	92%	97%	99%	99%	99%	99%	99%	99%	98%
Republic of Congo	93%	98%	99%	99%	99%	99%	99%	99%	98%
Rwanda	97%	99%	100%	100%	100%	99%	99%	99%	98%
Sao Tome and Principe	98%	99%	100%	100%	100%	100%	99%	99%	99%
Senegal	97%	99%	100%	100%	100%	99%	99%	99%	98%
Sierra Leone	89%	96%	99%	99%	99%	99%	98%	98%	97%
Somalia	90%	96%	99%	99%	99%	98%	98%	97%	96%
South Africa	97%	99%	100%	100%	99%	98%	97%	97%	96%
South Sudan	93%	97%	99%	99%	99%	99%	99%	98%	98%
Sudan	96%	99%	100%	100%	100%	100%	99%	99%	99%
Tanzania	96%	99%	99%	100%	99%	99%	99%	99%	98%
Togo	95%	98%	99%	99%	99%	99%	99%	98%	98%
Tunisia	99%	100%	100%	100%	100%	100%	100%	100%	99%
Uganda	97%	99%	99%	100%	99%	99%	99%	99%	98%
Zambia	95%	98%	100%	100%	99%	99%	98%	98%	97%
Zimbabwe	96%	99%	99%	99%	99%	99%	98%	97%	96%

3.3 Country Age-specific General Survival Rates (45-85+ years)

Table 9: The table below contains Country Age-specific General Survival Rates for females aged 45 and above as obtained from the WHO Global Health Observatory (Africa, 2020) [2]

COUNTRY	45-49	50-54	55-59	60-64	65-69	70-74	75-79	80-84	85+
Algeria	99%	99%	98%	97%	95%	92%	86%	75%	84%
Angola	97%	96%	95%	92%	89%	84%	77%	66%	72%
Benin	98%	97%	95%	93%	89%	84%	76%	66%	74%
Botswana	95%	94%	93%	91%	88%	83%	76%	65%	74%
Burkina Faso	98%	96%	95%	92%	89%	83%	75%	65%	76%
Burundi	97%	96%	94%	91%	88%	82%	74%	64%	74%
Cabo Verde	99%	98%	98%	96%	94%	90%	84%	72%	83%
Cameroon	96%	95%	94%	92%	89%	84%	76%	66%	78%
Central African Republic	94%	92%	89%	86%	82%	76%	67%	55%	62%
Chad	97%	96%	94%	91%	87%	81%	73%	63%	74%
Comoros	98%	97%	95%	93%	90%	84%	77%	66%	78%
Congo	96%	95%	93%	90%	87%	81%	73%	62%	70%
Côte d'Ivoire	97%	96%	95%	93%	89%	84%	77%	67%	79%
Djibouti	97%	96%	95%	93%	90%	85%	77%	67%	78%
Egypt	99%	98%	96%	94%	89%	85%	79%	69%	79%
Equatorial Guinea	96%	95%	94%	93%	90%	85%	77%	66%	76%
Eritrea	97%	95%	93%	90%	87%	81%	72%	61%	68%
Eswatini	95%	94%	93%	91%	88%	83%	75%	63%	71%
Ethiopia	98%	97%	96%	94%	91%	86%	78%	67%	79%
Gabon	97%	96%	95%	93%	91%	86%	78%	67%	77%
Gambia	97%	96%	94%	92%	89%	83%	75%	64%	73%
Ghana	97%	96%	95%	93%	90%	85%	77%	67%	78%
Guinea	97%	95%	93%	91%	87%	81%	73%	63%	73%

Guinea-Bissau	96%	95%	93%	90%	86%	80%	72%	61%	70%
Kenya	97%	96%	95%	93%	90%	85%	78%	67%	77%
Lesotho	90%	89%	88%	86%	83%	77%	69%	57%	63%
Liberia	97%	96%	94%	92%	88%	83%	75%	65%	76%
Libya	99%	98%	97%	95%	92%	89%	84%	77%	93%
Madagascar	97%	96%	94%	91%	87%	82%	74%	63%	72%
Malawi	97%	96%	95%	93%	90%	85%	77%	67%	79%
Mali	97%	96%	94%	92%	88%	82%	73%	63%	74%
Mauritania	98%	97%	96%	93%	90%	85%	77%	66%	77%
Mauritius	99%	98%	97%	96%	93%	90%	84%	76%	88%
Morocco	99%	98%	96%	94%	91%	86%	79%	68%	77%
Mozambique	95%	94%	93%	91%	88%	83%	75%	64%	74%
Namibia	96%	95%	94%	93%	91%	86%	78%	67%	77%
Niger	97%	96%	95%	92%	88%	82%	74%	64%	75%
Nigeria	98%	97%	95%	93%	90%	85%	76%	66%	78%
Republic of Congo	97%	96%	94%	92%	88%	83%	75%	64%	74%
Rwanda	98%	97%	96%	94%	90%	85%	78%	67%	78%
Sao Tome and Principe	98%	97%	96%	94%	90%	85%	76%	65%	72%
Senegal	98%	97%	96%	93%	90%	85%	77%	67%	78%
Sierra Leone	96%	94%	92%	89%	85%	79%	71%	61%	70%
Somalia	95%	93%	89%	85%	80%	74%	65%	55%	62%
South Africa	96%	96%	95%	94%	91%	88%	83%	73%	82%
South Sudan	97%	96%	95%	93%	90%	85%	77%	67%	79%
Sudan	98%	98%	96%	94%	91%	86%	78%	67%	78%
Tanzania	98%	97%	95%	93%	90%	85%	77%	67%	79%
Togo	97%	96%	95%	93%	89%	84%	77%	66%	78%
Tunisia	99%	99%	98%	97%	95%	91%	86%	76%	89%
Uganda	98%	97%	96%	94%	91%	86%	78%	67%	80%
Zambia	96%	95%	94%	92%	88%	83%	76%	65%	75%

Zimbabwe	95%	94%	92%	90%	87%	81%	73%	62%	70%

3.4 Country Age-specific Cancer Survival Rates (0-44 years)

Table 10: The table below contains country age-specific cancer survival rates for females aged 0-44 as obtained from the WHO Global Health Observatory (Africa, 2020) [2]

COUNTRY	0-4	5-9	10-14	15-19	20-24	25-29	30-34	35-39	40-44
Algeria	41%	56%	71%	57%	50%	45%	52%	56%	59%
Angola	39%	61%	77%	63%	42%	42%	47%	49%	50%
Benin	30%	42%	42%	47%	32%	31%	34%	36%	37%
Botswana	40%	67%	56%	65%	59%	49%	53%	56%	59%
Burkina Faso	28%	36%	48%	39%	33%	26%	32%	31%	33%
Burundi	23%	41%	47%	40%	29%	28%	32%	33%	34%
Cabo Verde	40%	75%	0%	74%	46%	49%	49%	61%	63%
Cameroon	35%	69%	64%	56%	43%	41%	46%	48%	50%
Central African Republic	21%	40%	37%	37%	25%	23%	26%	28%	28%
Chad	24%	32%	42%	40%	27%	27%	31%	32%	33%
Comoros	34%	65%	54%	54%	39%	40%	44%	43%	45%
Congo	26%	45%	53%	44%	33%	29%	31%	32%	34%
Côte d'Ivoire	35%	37%	59%	52%	38%	36%	41%	41%	44%
Djibouti	24%	73%	50%	40%	29%	28%	33%	35%	39%
Egypt	36%	52%	67%	59%	48%	43%	48%	50%	51%
Equatorial Guinea	37%	66%	60%	75%	42%	43%	49%	50%	52%
Eritrea	19%	30%	40%	27%	19%	18%	23%	24%	23%
Eswatini	34%	53%	46%	44%	33%	34%	35%	39%	42%
Ethiopia	29%	49%	51%	39%	32%	32%	34%	36%	37%
Gabon	44%	69%	55%	57%	47%	48%	55%	57%	56%
Gambia	35%	79%	69%	59%	43%	44%	48%	47%	49%
Ghana	33%	47%	56%	55%	41%	36%	41%	41%	42%

Guinea	31%	66%	46%	48%	37%	36%	38%	38%	39%
Guinea-Bissau	30%	34%	39%	43%	33%	31%	33%	35%	37%
Kenya	26%	44%	50%	41%	40%	33%	35%	36%	38%
Lesotho	31%	47%	53%	41%	31%	34%	38%	40%	42%
Liberia	31%	37%	49%	52%	38%	33%	37%	38%	40%
Libya	40%	60%	75%	62%	65%	52%	56%	57%	60%
Madagascar	33%	56%	59%	52%	39%	36%	39%	39%	41%
Malawi	27%	58%	45%	43%	30%	28%	34%	31%	32%
Mali	28%	36%	52%	45%	30%	28%	31%	32%	33%
Mauritania	33%	48%	49%	55%	37%	35%	38%	40%	42%
Mauritius	45%	60%	69%	79%	56%	56%	56%	53%	63%
Morocco	37%	61%	66%	54%	51%	45%	47%	50%	52%
Mozambique	30%	33%	46%	48%	33%	32%	35%	33%	34%
Namibia	50%	67%	80%	77%	63%	62%	68%	68%	69%
Niger	26%	45%	40%	47%	30%	27%	29%	29%	30%
Nigeria	32%	30%	50%	44%	39%	36%	38%	38%	41%
Republic of Congo	36%	52%	66%	61%	44%	38%	42%	44%	45%
Rwanda	29%	40%	53%	45%	39%	33%	36%	39%	39%
Sao Tome and Principe	40%	0%	42%	65%	64%	40%	41%	41%	49%
Senegal	31%	42%	54%	51%	33%	33%	36%	38%	39%
Sierra Leone	29%	29%	49%	51%	29%	28%	32%	34%	35%
Somalia	21%	33%	37%	29%	23%	23%	25%	25%	25%
South Africa	38%	57%	66%	59%	47%	45%	51%	53%	53%
South Sudan	22%	38%	42%	34%	25%	24%	27%	27%	27%
Sudan	29%	40%	53%	44%	34%	32%	34%	36%	38%
Tanzania	27%	48%	57%	47%	40%	35%	37%	37%	39%
Togo	30%	61%	42%	45%	33%	32%	35%	36%	38%
Tunisia	40%	63%	76%	59%	56%	52%	56%	61%	64%
Uganda	28%	46%	50%	47%	37%	37%	40%	39%	40%

Zambia	35%	48%	57%	56%	46%	43%	45%	45%	47%
Zimbabwe	38%	61%	71%	61%	52%	46%	51%	50%	54%

3.5 Country Age-specific Cancer Survival Rates (45-85+ years)

Table 11: The table below contains country age-specific cancer survival rates for females aged 45 and above as obtained from the WHO Global Health Observatory (Africa, 2020) [2]

COUNTRY	45-49	50-54	55-59	60-64	65-69	70-74	75-79	80-84	85+
Algeria	61%	65%	64%	59%	63%	59%	52%	49%	46%
Angola	54%	54%	52%	55%	50%	48%	45%	43%	42%
Benin	39%	41%	39%	39%	36%	32%	30%	24%	17%
Botswana	63%	60%	56%	61%	61%	58%	57%	51%	43%
Burkina Faso	33%	34%	33%	34%	33%	32%	31%	28%	23%
Burundi	32%	33%	31%	33%	35%	31%	28%	26%	22%
Cabo Verde	63%	63%	69%	62%	64%	39%	37%	40%	40%
Cameroon	52%	55%	52%	51%	48%	45%	41%	35%	26%
Central African Republic	30%	33%	30%	31%	31%	28%	27%	24%	20%
Chad	35%	36%	34%	33%	35%	32%	30%	27%	22%
Comoros	47%	49%	45%	46%	46%	39%	40%	39%	44%
Congo	36%	39%	37%	37%	35%	34%	32%	28%	23%
Côte d'Ivoire	46%	48%	45%	45%	45%	42%	40%	37%	31%
Djibouti	40%	42%	40%	41%	37%	37%	37%	29%	42%
Egypt	52%	52%	49%	48%	44%	48%	43%	39%	35%
Equatorial Guinea	50%	56%	53%	50%	50%	46%	40%	39%	0%
Eritrea	30%	27%	27%	25%	25%	25%	24%	23%	18%
Eswatini	43%	42%	36%	38%	36%	37%	37%	33%	29%
Ethiopia	39%	41%	38%	39%	37%	37%	36%	32%	27%
Gabon	59%	62%	60%	56%	53%	51%	45%	38%	33%

Gambia	48%	52%	51%	51%	46%	45%	45%	41%	35%
Ghana	44%	48%	46%	47%	42%	40%	36%	30%	23%
Guinea	38%	39%	37%	38%	40%	37%	35%	31%	24%
Guinea-Bissau	38%	38%	36%	34%	37%	36%	34%	27%	19%
Kenya	40%	41%	38%	38%	38%	37%	32%	27%	22%
Lesotho	40%	42%	39%	41%	39%	38%	36%	28%	25%
Liberia	41%	43%	40%	41%	42%	38%	38%	36%	28%
Libya	64%	69%	67%	62%	62%	55%	48%	41%	32%
Madagascar	43%	45%	43%	43%	44%	40%	37%	34%	31%
Malawi	33%	35%	33%	33%	31%	31%	31%	30%	28%
Mali	35%	37%	34%	34%	34%	31%	31%	28%	22%
Mauritania	43%	45%	43%	43%	40%	39%	36%	32%	28%
Mauritius	62%	63%	68%	65%	67%	72%	62%	53%	50%
Morocco	55%	57%	55%	56%	59%	49%	46%	43%	37%
Mozambique	35%	36%	35%	34%	33%	32%	31%	28%	23%
Namibia	72%	73%	71%	71%	73%	68%	63%	63%	52%
Niger	32%	34%	33%	32%	30%	29%	28%	25%	18%
Nigeria	43%	45%	42%	43%	42%	41%	39%	35%	28%
Republic of Congo	48%	51%	47%	48%	46%	44%	43%	38%	30%
Rwanda	41%	37%	39%	39%	39%	38%	39%	29%	30%
Sao Tome and Principe	47%	36%	32%	50%	49%	35%	37%	35%	0%
Senegal	40%	42%	40%	39%	39%	37%	35%	32%	27%
Sierra Leone	37%	38%	37%	36%	35%	34%	32%	29%	20%
Somalia	27%	29%	28%	27%	27%	27%	25%	23%	18%
South Africa	54%	56%	54%	53%	55%	54%	50%	46%	41%
South Sudan	28%	30%	29%	30%	26%	26%	25%	22%	19%
Sudan	40%	43%	41%	40%	40%	39%	36%	33%	29%
Tanzania	40%	42%	39%	40%	37%	36%	35%	31%	27%
Togo	40%	42%	39%	39%	38%	36%	34%	29%	23%

Tunisia	63%	69%	69%	66%	69%	64%	53%	47%	44%
Uganda	40%	42%	39%	39%	38%	36%	35%	28%	23%
Zambia	49%	49%	46%	46%	44%	42%	40%	36%	29%
Zimbabwe	56%	57%	53%	52%	53%	47%	44%	39%	29%

3.6 Country Age-specific Relative Survival Rates (0-44 years)

Table 12: The table below contains country age-specific relative survival rates for females aged 0-44 as computed by the authors (Africa, 2020).

COUNTRY	0-4	5-9	10-14	15-19	20-24	25-29	30-34	35-39	40-44
Algeria	42%	56%	71%	57%	50%	45%	52%	57%	60%
Angola	42%	62%	78%	63%	42%	42%	48%	50%	51%
Benin	32%	43%	42%	47%	32%	31%	35%	37%	38%
Botswana	41%	68%	57%	65%	59%	49%	54%	58%	62%
Burkina Faso	30%	37%	48%	39%	33%	26%	32%	32%	34%
Burundi	24%	42%	47%	40%	29%	28%	32%	34%	35%
Cabo Verde	41%	75%	0%	74%	46%	49%	50%	61%	63%
Cameroon	37%	70%	65%	57%	43%	41%	46%	49%	51%
Central African Republic	23%	41%	37%	37%	26%	23%	27%	29%	29%
Chad	27%	33%	42%	40%	27%	28%	31%	32%	33%
Comoros	36%	65%	54%	54%	40%	40%	44%	44%	46%
Congo	27%	45%	53%	45%	33%	29%	31%	32%	35%
Côte d'Ivoire	37%	37%	60%	52%	38%	36%	42%	42%	45%
Djibouti	25%	73%	50%	40%	29%	29%	33%	36%	40%
Egypt	37%	52%	67%	59%	48%	43%	48%	50%	52%
Equatorial Guinea	39%	67%	61%	75%	42%	44%	50%	52%	54%
Eritrea	19%	30%	40%	27%	19%	18%	24%	25%	23%
Eswatini	35%	54%	47%	45%	33%	34%	36%	41%	44%
Ethiopia	30%	50%	51%	39%	32%	32%	35%	37%	38%

Gabon	46%	70%	55%	57%	47%	48%	55%	58%	58%
Gambia	36%	80%	69%	59%	44%	45%	48%	48%	51%
Ghana	34%	47%	56%	55%	41%	36%	41%	41%	43%
Guinea	33%	68%	47%	49%	37%	37%	39%	39%	40%
Guinea-Bissau	31%	35%	40%	43%	33%	31%	34%	36%	38%
Kenya	27%	45%	51%	41%	40%	33%	36%	37%	39%
Lesotho	33%	47%	54%	42%	32%	35%	40%	43%	45%
Liberia	33%	37%	49%	52%	38%	33%	37%	39%	41%
Libya	40%	60%	75%	62%	66%	52%	56%	58%	61%
Madagascar	34%	57%	59%	53%	39%	36%	39%	39%	42%
Malawi	28%	58%	45%	43%	30%	28%	34%	32%	33%
Mali	30%	37%	53%	45%	30%	28%	31%	32%	34%
Mauritania	35%	49%	49%	55%	37%	35%	38%	40%	43%
Mauritius	45%	60%	69%	79%	57%	56%	57%	53%	64%
Morocco	38%	61%	66%	54%	51%	45%	47%	51%	52%
Mozambique	31%	34%	46%	48%	33%	33%	36%	35%	36%
Namibia	52%	68%	80%	77%	63%	63%	69%	69%	71%
Niger	27%	47%	40%	48%	30%	27%	29%	30%	31%
Nigeria	34%	31%	50%	44%	39%	36%	38%	39%	41%
Republic of Congo	38%	53%	67%	62%	44%	38%	42%	45%	46%
Rwanda	30%	40%	53%	45%	39%	33%	36%	40%	40%
Sao Tome and Principe	41%	0%	42%	65%	64%	40%	41%	41%	49%
Senegal	32%	42%	54%	51%	33%	33%	36%	38%	40%
Sierra Leone	33%	30%	49%	51%	29%	29%	33%	35%	36%
Somalia	23%	35%	37%	30%	23%	23%	26%	25%	26%
South Africa	39%	57%	66%	59%	47%	46%	52%	55%	55%
South Sudan	23%	40%	42%	34%	25%	24%	27%	27%	28%
Sudan	31%	41%	53%	44%	34%	32%	34%	37%	38%
Tanzania	28%	49%	57%	47%	40%	35%	38%	37%	39%

Togo	31%	63%	42%	46%	33%	32%	35%	36%	39%
Tunisia	40%	63%	77%	59%	56%	52%	57%	61%	64%
Uganda	29%	47%	50%	47%	37%	37%	41%	40%	41%
Zambia	36%	48%	57%	57%	46%	43%	46%	46%	48%
Zimbabwe	40%	62%	71%	62%	52%	46%	52%	52%	56%

3.7 Country Age-specific Relative Survival Rates (45-85+ years)

Table 13: The table below contains country age-specific relative survival rates for females aged 45 and above as computed by the authors (Africa, 2020).

COUNTRY	45-49	50-54	55-59	60-64	65-69	70-74	75-79	80-84	85+
Algeria	62%	66%	65%	61%	66%	64%	60%	65%	55%
Angola	56%	56%	55%	59%	56%	58%	59%	65%	58%
Benin	40%	42%	41%	41%	40%	39%	40%	37%	24%
Botswana	66%	64%	61%	67%	70%	70%	76%	79%	58%
Burkina Faso	34%	35%	35%	37%	37%	38%	41%	43%	31%
Burundi	33%	34%	33%	36%	40%	38%	38%	41%	29%
Cabo Verde	64%	64%	71%	64%	68%	43%	44%	55%	49%
Cameroon	54%	58%	55%	55%	54%	54%	54%	53%	34%
Central African Republic	32%	36%	34%	35%	37%	37%	40%	43%	32%
Chad	36%	38%	36%	37%	40%	39%	41%	43%	30%
Comoros	48%	51%	47%	50%	51%	47%	53%	60%	56%
Congo	38%	41%	39%	41%	41%	41%	43%	46%	32%
Côte d'Ivoire	47%	50%	47%	49%	51%	50%	52%	56%	39%
Djibouti	41%	44%	42%	44%	41%	44%	48%	44%	54%
Egypt	53%	53%	51%	52%	50%	57%	55%	56%	44%
Equatorial Guinea	52%	58%	56%	54%	55%	54%	52%	59%	0%
Eritrea	31%	28%	28%	28%	29%	31%	33%	37%	27%

Eswatini	45%	44%	39%	41%	41%	44%	49%	52%	41%
Ethiopia	40%	42%	40%	41%	41%	43%	46%	47%	34%
Gabon	60%	64%	63%	60%	59%	59%	57%	56%	43%
Gambia	50%	54%	54%	56%	52%	54%	60%	64%	48%
Ghana	46%	50%	49%	51%	46%	47%	46%	45%	29%
Guinea	40%	41%	39%	41%	46%	46%	48%	50%	33%
Guinea-Bissau	39%	40%	38%	38%	43%	45%	47%	44%	27%
Kenya	41%	43%	40%	40%	42%	44%	41%	41%	29%
Lesotho	44%	47%	44%	47%	47%	49%	52%	50%	39%
Liberia	42%	44%	43%	45%	47%	46%	50%	55%	38%
Libya	65%	71%	69%	65%	67%	62%	57%	53%	34%
Madagascar	44%	47%	45%	47%	50%	49%	51%	55%	42%
Malawi	34%	36%	35%	36%	34%	36%	40%	45%	35%
Mali	36%	39%	37%	37%	39%	38%	42%	44%	30%
Mauritania	44%	46%	45%	46%	45%	46%	47%	49%	37%
Mauritius	63%	65%	70%	67%	72%	80%	74%	70%	56%
Morocco	56%	59%	57%	59%	65%	57%	58%	63%	48%
Mozambique	37%	39%	37%	38%	37%	39%	41%	43%	31%
Namibia	75%	77%	75%	77%	80%	79%	81%	93%	67%
Niger	33%	35%	35%	35%	34%	36%	37%	39%	24%
Nigeria	44%	46%	44%	46%	46%	49%	51%	53%	35%
Republic of Congo	49%	54%	50%	52%	52%	53%	57%	59%	41%
Rwanda	42%	39%	41%	42%	43%	44%	51%	44%	38%
Sao Tome and Principe	48%	37%	33%	53%	54%	41%	49%	53%	0%
Senegal	41%	43%	42%	42%	43%	43%	45%	48%	34%
Sierra Leone	39%	41%	40%	41%	41%	43%	46%	48%	29%
Somalia	29%	31%	31%	32%	34%	37%	38%	42%	29%
South Africa	56%	59%	57%	57%	60%	62%	61%	63%	50%
South Sudan	29%	31%	30%	32%	28%	31%	33%	33%	23%

Sudan	41%	44%	43%	43%	44%	45%	47%	49%	37%
Tanzania	41%	43%	41%	42%	41%	42%	45%	47%	34%
Togo	41%	44%	41%	42%	42%	43%	45%	44%	30%
Tunisia	63%	70%	70%	68%	73%	70%	62%	62%	50%
Uganda	41%	43%	41%	41%	42%	42%	45%	42%	29%
Zambia	50%	52%	49%	50%	50%	50%	53%	55%	39%
Zimbabwe	58%	60%	57%	58%	61%	57%	60%	63%	42%

4 Incidence Metadata Sources

Table 14: This table provided the sources used to obtain cancer incidence data for the respective countries (Africa, 2020)

Country	2016	2017	2018	2019	2020
Algeria	Estimated from Age-specific population structure	Estimated from Age-specific population structure	[2]	Estimated from Age-specific population structure	[1]
Angola	Estimated from Age-specific population structure	Estimated from Age-specific population structure	[2]	Estimated from Age-specific population structure	[1]
Benin	Estimated from Age-specific population structure	Estimated from Age-specific population structure	[2]	Estimated from Age-specific population structure	[1]
Botswana	Estimated from Age-specific population structure	Estimated from Age-specific population structure	[2]	Estimated from Age-specific population structure	[1]
Burkina Faso	Estimated from Age-specific population structure	Estimated from Age-specific population structure	[2]	Estimated from Age-specific population structure	[1]

Burundi	Estimated from Age-specific population structure	Estimated from Age-specific population structure	[2]	Estimated from Age-specific population structure	[1]
Cabo Verde	Estimated from Age-specific population structure	Estimated from Age-specific population structure	[2]	Estimated from Age-specific population structure	[1]
Cameroon	Estimated from Age-specific population structure	Estimated from Age-specific population structure	[2]	Estimated from Age-specific population structure	[1]
Central African Republic	Estimated from Age-specific population structure	Estimated from Age-specific population structure	[2]	Estimated from Age-specific population structure	[1]
Chad	Estimated from Age-specific population structure	Estimated from Age-specific population structure	[2]	Estimated from Age-specific population structure	[1]

Comoros	Estimated from Age-specific population structure	Estimated from Age-specific population structure	[2]	Estimated from Age-specific population structure	[1]
Congo	Estimated from Age-specific population structure	Estimated from Age-specific population structure	[2]	Estimated from Age-specific population structure	[1]
Côte d'Ivoire	Estimated from Age-specific population structure	Estimated from Age-specific population structure	[2]	Estimated from Age-specific population structure	[1]
Djibouti	Estimated from Age-specific population structure	Estimated from Age-specific population structure	[2]	Estimated from Age-specific population structure	[1]
Egypt	Estimated from Age-specific population structure	Estimated from Age-specific population structure	[2]	Estimated from Age-specific population structure	[1]

Equatorial Guinea	Estimated from Age-specific population structure	Estimated from Age-specific population structure	[2]	Estimated from Age-specific population structure	[1]
Eritrea	Estimated from Age-specific population structure	Estimated from Age-specific population structure	[2]	Estimated from Age-specific population structure	[1]
Eswatini	Estimated from Age-specific population structure	Estimated from Age-specific population structure	[2]	Estimated from Age-specific population structure	[1]
Ethiopia	[3]	Estimated from Age-specific population structure	[2]	Estimated from Age-specific population structure	[1]
Gabon	Estimated from Age-specific population structure	Estimated from Age-specific population structure	[2]	Estimated from Age-specific population structure	[1]

Gambia	Estimated from Age-specific population structure	Estimated from Age-specific population structure	[2]	Estimated from Age-specific population structure	[1]
Ghana	Estimated from Age-specific population structure	Estimated from Age-specific population structure	[2]	Estimated from Age-specific population structure	[1]
Guinea	Estimated from Age-specific population structure	Estimated from Age-specific population structure	[4]	Estimated from Age-specific population structure	[1]
Guinea-Bissau	Estimated from Age-specific population structure	Estimated from Age-specific population structure	[2]	Estimated from Age-specific population structure	[1]
Kenya	Estimated from Age-specific population structure	Estimated from Age-specific population structure	[5]	Estimated from Age-specific population structure	[1]

Lesotho	Estimated from Age-specific population structure	Estimated from Age-specific population structure	[2]	Estimated from Age-specific population structure	[1]
Liberia	Estimated from Age-specific population structure	Estimated from Age-specific population structure	[2]	Estimated from Age-specific population structure	[1]
Libya	Estimated from Age-specific population structure	Estimated from Age-specific population structure	[2]	Estimated from Age-specific population structure	[1]
Madagascar	Estimated from Age-specific population structure	Estimated from Age-specific population structure	[2]	Estimated from Age-specific population structure	[1]
Malawi	Estimated from Age-specific population structure	Estimated from Age-specific population structure	[2]	Estimated from Age-specific population structure	[1]

Mali	Estimated from Age-specific population structure	Estimated from Age-specific population structure	[2]	Estimated from Age-specific population structure	[1]
Mauritania	Estimated from Age-specific population structure	Estimated from Age-specific population structure	[2]	Estimated from Age-specific population structure	[1]
Mauritius	Estimated from Age-specific population structure	[6]	[6]	[7]	[1]
Morocco	Estimated from Age-specific population structure	Estimated from Age-specific population structure	[2]	Estimated from Age-specific population structure	[1]
Mozambique	Estimated from Age-specific population structure	Estimated from Age-specific population structure	[2]	Estimated from Age-specific population structure	[1]

Namibia	Estimated from Age-specific population structure	Estimated from Age-specific population structure	[2]	Estimated from Age-specific population structure	[1]
Niger	Estimated from Age-specific population structure	Estimated from Age-specific population structure	[2]	Estimated from Age-specific population structure	[1]
Nigeria	Estimated from Age-specific population structure	Estimated from Age-specific population structure	[4]	Estimated from Age-specific population structure	[1]
Republic of Congo	Estimated from Age-specific population structure	Estimated from Age-specific population structure	[2]	Estimated from Age-specific population structure	[1]
Rwanda	Estimated from Age-specific population structure	[8]	[9]	Estimated from Age-specific population structure	[1]

Sao Tome and Principe	Estimated from Age-specific population structure	Estimated from Age-specific population structure	[2]	Estimated from Age-specific population structure	[1]
Senegal	Estimated from Age-specific population structure	Estimated from Age-specific population structure	[2]	Estimated from Age-specific population structure	[1]
Sierra Leone	Estimated from Age-specific population structure	Estimated from Age-specific population structure	[2]	Estimated from Age-specific population structure	[1]
Somalia	Estimated from Age-specific population structure	Estimated from Age-specific population structure	[2]	Estimated from Age-specific population structure	[1]
South Africa	[10]	[11]	[12]	[13]	[1]
South Sudan	Estimated from Age-specific population structure	Estimated from Age-specific population structure	[2]	Estimated from Age-specific population structure	[1]

Sudan	Estimated from Age-specific population structure	Estimated from Age-specific population structure	[2]	Estimated from Age-specific population structure	[1]
Tanzania	Estimated from Age-specific population structure	Estimated from Age-specific population structure	[2]	Estimated from Age-specific population structure	[1]
Togo	Estimated from Age-specific population structure	Estimated from Age-specific population structure	[2]	Estimated from Age-specific population structure	[1]
Tunisia	Estimated from Age-specific population structure	Estimated from Age-specific population structure	[2]	Estimated from Age-specific population structure	[1]
Uganda	[14]	[14]	[4]	Estimated from Age-specific population structure	[1]

Zambia	Estimated from Age-specific population structure	Estimated from Age-specific population structure	[2]	Estimated from Age-specific population structure	[1]
Zimbabwe	[15]	[16]	[2]	Estimated from Age-specific population structure	[1]

5 Analysis of Variance

Table 15: Tests of Homogeneity of Variances as obtained from SPSS output (Africa, 2020)

	**Levene Statistic**	**df1**	**df2**	**Sig.**
Based on Mean	0.261	4	85	0.902
Based on Median	0.153	4	85	0.961
Based on Median and with adjusted df	0.153	4	77.794	0.961
Based on trimmed mean	0.229	4	85	0.921

Table 16: Post Hoc Tests: Tukey HSD as obtained from SPSS output (Africa, 2020)

Region (I)	Region (J)	Mean Diff. (I-J)	Std. Error	Sig.	95% CI	
					Lower	Upper
W/Africa	E/Africa	0.00534	0.02132	0.999	-0.0541	0.0648
	N/Africa	-0.12414*	0.02132	0.000	-0.1836	-0.0647
	S/Africa	-0.13368*	0.02132	0.000	-0.1931	-0.0743
	C/Africa	-0.03839	0.02132	0.380	-0.0978	0.0210
E/Africa	W/Africa	-0.00534	0.02132	0.999	-0.0648	0.0541
	N/Africa	-0.12948*	0.02132	0.000	-0.1889	-0.0701
	S/Africa	-0.13902*	0.02132	0.000	-0.1985	-0.0796
	C/Africa	-0.04373	0.02132	0.251	-0.1032	0.0157
N/Africa	W/Africa	0.12414*	0.02132	0.000	0.0647	0.1836
	E/Africa	0.12948*	0.02132	0.000	0.0701	0.1889
	S/Africa	-0.00954	0.02132	0.992	-0.0690	0.0499
	C/Africa	0.08575*	0.02132	0.001	0.0263	0.1452
S/Africa	W/Africa	0.13368*	0.02132	0.000	0.0743	0.1931
	E/Africa	0.13903*	0.02132	0.000	0.0796	0.1985
	N/Africa	0.00954	0.02132	0.992	-0.0499	0.0690
	C/Africa	0.09529*	0.02132	0.000	0.0359	0.1547
C/Africa	W/Africa	0.03839	0.02132	0.380	-0.0210	0.0978
	E/Africa	0.04373	0.02132	0.251	-0.0157	0.1032
	N/Africa	-0.08575*	0.02132	0.001	-0.1452	-0.0263
	S/Africa	-0.09529*	0.02132	0.000	-0.1547	-0.0359

*. The mean difference is significant at the 0.05 level.

Bibliography

[1] International Agency for Research on Cancer World Health Organization. *CANCER TODAY: Data visualization tools for exploring the global cancer burden in 2020.* Last accessed 10.07.2022. 2021. URL: https://gco.iarc.fr/today/.

[2] World Health Organization. *Global Health Observatory. Geneva: World Health Organization.* Last accessed 10.07.2019. 2018. URL: https://gco.iarc.fr/today/data-sources-methods#cancer-dictionary.

[3] Disease Prevention and Control Directorate of the Federal Ministry of Health (Ethiopia). *NATIONAL CANCER CONTROL PLAN (2016-2020).* 2015.

[4] Jacques Ferlay et al. 'Estimating the global cancer incidence and mortality in 2018: GLOBOCAN sources and methods'. In: *International journal of cancer* 144.8 (2019), pp. 1941–1953.

[5] Ministry of Health (Kenya). *KENYA CANCER POLICY (2019-2030).* 2018.

[6] Ministry of Health and Wellness (Mauritius); World Health Organization; Mauritius Institute of Health. *Cancer in the Republic of Mauritius: Incidence and Mortality Study for 2018 (Report of the National Cancer Registry).* 2019.

[7] Ministry of Health and Wellness (Mauritius); World Health Organization; African Cancer Registry Network. *Cancer in the Republic of Mauritius: Incidence and Mortality Study for 2020 (Report of the National Cancer Registry).* 2022.

[8] Michel Nkurunziza (The NewTimes newspaper). *Researchers record over 10,000 new cancer cases.* Last accessed: 24.12.22. 2019. URL: https://www.newtimes.co.rw/article/168892/News/researchers-record-over-10000-new-cancer-cases.

[9] Federal Ministry of Health (Republic of Rwanda). *RWANDA NATIONAL CANCER CONTROL PLAN (2020-2024).* 2020.

[10] National Health Laboratory Service (South Africa). *National Cancer Register 2016.* 2016.

[11] National Health Laboratory Service (South Africa). *National Cancer Register 2017*. 2017.

[12] National Health Laboratory Service (South Africa). *National Cancer Register 2018*. 2018.

[13] National Health Laboratory Service (South Africa). *National Cancer Register 2019*. 2019.

[14] Uganda Bureau of Statistics. *2020 Statistical Abstract*. 2020.

[15] ZimFact (Online fact-checking site). *Are cancer cases on the rise in Zimbabwe? Here's the data*. Last accessed: 24.12.22. 2020. URL: https://zimfact.org/are-cancer-cases-on-the-rise-in-zimbabwe-heres-the-data/.

[16] Environment & Innovations Editor of The Herald newspaper) Sifelani Tsiko (Agric. *Cancer cases rise as diet changes*. Last accessed: 24.12.22. 2021. URL: https://www.herald.co.zw/cancer-cases-rise-as-diet-changes/.
